# Behaviors and Risks for Cardiovascular Disease Among Muslim Women in the United States

**DOI:** 10.1089/heq.2018.0050

**Published:** 2018-10-08

**Authors:** Henna Budhwani, Seth Borgstede, Aarin L. Palomares, Roman B. Johnson, Kristine R. Hearld

**Affiliations:** ^1^ Department of Health Care Organization and Policy, University of Alabama at Birmingham, Birmingham, Alabama.; ^2^ Department of Sociology, University of Alabama at Birmingham, Birmingham, Alabama.; ^3^ Department of Health Services Administration, University of Alabama at Birmingham, Birmingham, Alabama.

**Keywords:** cardiovascular, heart disease, Muslim, Islam, women's health

## Abstract

**Purpose:** This study examines statistical associates of cardiovascular disease risk factors, as defined by the American Heart Association's Life's Simple Seven, among Muslim women who reside in the United States.

**Methods:** Data collected nationally through the 2015 Muslim Women's Health project were analyzed (*N*=373). Logistic regression models estimated associations between sample characteristics and diet, exercise, alcohol consumption, blood pressure, cholesterol, and weight.

**Results:** Over half of respondents reported exercising regularly (64%) and maintaining a healthy diet (85%); 20% consumed alcohol. About 5% reported having high cholesterol, 4% had high blood pressure, and 42% reported being overweight. Perceived and experienced stigma were associated with alcohol use (odds ratio [OR]=1.085, *p*<0.001) and being overweight (OR=0.938, *p*<0.001). Married respondents had 42% lower odds of exercising and 83% lower odds of drinking alcohol. Compared to foreign-born respondents, U.S.-born respondents had 2.9 higher odds of drinking alcohol and 2.7 higher odds of having high cholesterol (OR=2.931, *p*<0.001; OR=2.732, *p*<0.01, respectively). Significant effects were also found when examining the statistical impact of of age, Islamic sect, and education on cardiovascular disease risk factors.

**Conclusion:** With increasing focus on precision medicine, personalized healthcare, and patient-centered medical homes (all interventions designed to promote disease prevention and assist in managing chronic health conditions) better understanding the health of understudied populations is imperative to the success of these interventions. Our findings suggest countervailing forces may affect the health of American Muslim women; therefore, additional studies with this hard-to-reach population are warranted and will be informative to improving overall population health in the United States, an overarching priority for both public health practitioners and medical providers.

## Introduction

Currently, about a third of the United States identifies as a racial or ethnic minority, with a significant proportion of these individuals, particularly those who identify as Hispanic, Asian, and Middle Eastern, being foreign born.^1–3^ As the number and percentage of minority Americans grow, understanding their health, health behaviors, and risk factors for disease becomes more urgent and makes a greater impact on overall population health.^1,4,5^ Muslim Americans are an understudied population that is difficult to reach due to a range of factors that include this group's experiences with ongoing stigma and discrimination.^6,7^ Regardless, it is still imperative that clinicians, policy makers, and public health practitioners understand and identify ways to improve this population's health and well-being, particularly considering the countervailing forces that could influence health behaviors.^6,7^ Prior studies on Muslim Americans have focused on mental health, stigma, and sociopolitical measures.^7–10^ Few studies have examined preventative health behaviors and risk factors for noncommunicable diseases in Muslim Americans, with even fewer evaluating these conditions in American Muslim women.

Bearing in mind the importance of reducing rates of noncommunicable diseases, namely cardiovascular diseases, through the identification of risk factors and promotion of health lifestyle behaviors, this study assesses associations between nativity (defined as being an immigrant compared to being born in the United States), sociodemographic characteristics, heightened vigilance (defined as experiences of stigma), and health behaviors and health risks informed by the American Heart Association's Life's Simple Seven^11^ among American Muslim women. These seven steps include managing blood pressure, controlling cholesterol, reducing blood sugar, increasing physical activity, improving diet, losing weight, and smoking cessation.^11^ This study will examine diet, blood pressure, cholesterol, physical activity, and being overweight (obesity).

A phenomenon known as the “healthy migrant effect” is particularly relevant to this study.^12–16^ It illustrates significantly improved health outcomes of immigrant groups compared to the native populations of industrialized nations.^12–16^ Previous studies report immigrant groups are shown to have significantly lower mortality rates from illnesses such as cardiovascular disease, HIV/AIDS, cirrhosis, diabetes, respiratory diseases, suicide, mental illness, and certain cancers when compared to their native American-born counterparts.^14–18^ An explanation for this phenomenon is self-selection in migration, in which healthier individuals are more likely to migrate. Thus, these groups could have different health behaviors and outcomes than nonmigrant groups in their country of origin. Although the literature documents improved health outcomes among immigrant groups, it also reveals that as migrants become more acculturated in industrialized nations, health status may decline.^18,19^ For example, immigrants living in the United States for <1 year were shown to have obesity rates of 8%, while immigrants living in the United States for >15 years show obesity rates of 19%.^20^ Because many noncommunicable diseases can be attributed to health behaviors, it is of particular importance to assess health behaviors of populations with large proportions of foreign-born members.

In terms of diet, upon arrival in the United States, Arab migrants—typically Muslim—engage in healthier eating patterns, ascribing to diets that resemble food from their country of origin; the longer immigrants reside in the United States, the more likely their eating patterns are to resemble that of a typical American diet.^18,21,22^ Religion also plays a significant role in the development of eating patterns, especially in Islam. Islam prohibits consumption of pork and alcohol with ∼75% of Muslims living in the United States reporting that they follow the dietary guidelines set forth by the Quran, whereas only 16% of Jewish households in the United States follow kosher guidelines.^23,24^ These recommendations from the Quran, if adhered to, can be significant contributors to better health outcomes among Muslim migrant groups.

Despite a healthier diet, the literature suggests a greater rate of physical inactivity among migrant groups.^25^ Studies revealed that South Asians have a higher risk of developing type 2 diabetes and coronary heart disease than people of European origin.^26^ Arab Muslim women groups, in particular, are shown to be less likely to engage in physical activity when adapting to a new culture due to varying factors, including shame, cultural pressures, and modesty.^27,28^ The prevalence of physical inactivity varies among subgroups, especially within Arab, Muslim, and women subgroups, and one study found that Muslim ethnicity was a significant statistical predictor for being physically inactive.^29^ Because the American Heart Association outlines getting active as one of the seven steps to establishing improved health outcomes,^11^ and immigrant groups are shown to be less likely to be physically active, but more likely to show improved health, further research is needed to understand why this paradox relationship exists.

These behaviors, namely diet and exercise, influence risk factors such as hypertension, high blood pressure, and high cholesterol, which in turn increase the likelihood of developing cardiovascular disease. Prevalence rates of hypertension are as high as 57.6% among immigrants compared to 31.7% among native-born individuals in the United States.^30^ Foreign-born South Asian women are at elevated risks of hypertension compared to their American-born counterparts,^31^ and a study on Arab Americans found that 76.2% of the respondents were either hypertensive or prehypertensive.^32^ As for cholesterol, multiple studies have found that immigrant populations have lower levels compared to their counterparts who were born in industrialized nations.^33–35^ A study conducted in the Netherlands showed total cholesterol levels among Moroccan and Turkish immigrants were significantly lower than the native Dutch populations. In women, differences in these cholesterol levels were even more notable with the Moroccan and Turkish women showing reductions of 25% and 40%, respectively, in high-density lipoprotein (HDL) cholesterol when compared to Dutch women.^33^ Other studies in Scandinavian nations demonstrated that immigrants from Sri Lanka and Pakistan had significantly lower HDL-cholesterol levels, but reported higher blood pressure, which reiterates that immigrants with healthy dietary habits, but poor physical activity, will likely demonstrate mixed outcomes, and that Turkish immigrant women demonstrated a significantly worse cardiovascular risk profile when compared to native women.^34,35^

Considering researchers and clinicians know little about preventative health behaviors and health risks of Muslim Americans, this study—which attempts to address this gap and initiate a dialogue on the health of American Muslim women—is both novel and timely, offering meaningful results for those seeking to improve population health through prevention.

## Methods

### Study design and participants

The Muslim Women's Health project was funded by the University of Alabama at Birmingham School of Public Health's Back of the Envelope mechanism. Online data collection occurred for 3 months between September 2015 and December 2015. Information on the Muslim Women's Health project, the principal investigator, the funding institution, ethical approval from the University of Alabama at Birmingham's Institutional Review Board (IRB) No. X150413001, and contact information were available at an online portal that was live through the duration of the data collection cycle. Informed consent was attained by all respondents through an IRB-approved online consent form. Women who self-identified as Muslim, were at least 18 years old, and were current residents of the United States were eligible to participate. Average time to complete the survey was <15 min. Although online surveys have limitations, such as sampling bias, one major benefit is the ability to connect with difficult-to-reach populations, which include those holding unpopular beliefs, small minority groups, and groups that are stigmatized.^36^ Due to the employment of snowball sampling without unique personal identifiers, a response rate could not be calculated. After removing missing data, the final sample size for this study was *n*=373.

### Measures

The analyses examined correlates of prevention health behaviors and health risks among Muslim women residing in the United States. Health behaviors are activities that are performed frequently and have an impact on an individual's daily life, requiring personal responsibility on the part of the individual to perform the activities. The three health behaviors of interest were as follows: (1) exercising, (2) healthy eating, and (3) limited alcohol consumption. Since the Office of Disease Prevention and Health Promotion has recommended that adults exercise for at least 30 min on 4 or more days a week, respondents who met these recommendations were coded as 1 and those who did not meet the recommendations were coded as 0. Respondents reporting eating fruit and vegetables daily were coded as 1 for healthy eating, while those who did not were recorded as 0. Respondents who reported that they currently drink alcohol were recorded as 1 and those who do not drink alcohol were recorded as 0. Also included are three health risks: (1) high blood pressure, (2) high cholesterol, and (3) being overweight as defined by body mass index (BMI). Respondents were asked if their blood pressure was high (>120/80), normal (∼90–120/60–80), or low (<90/60). Respondents with high blood pressure were coded as 1 and others were coded as 0. Likewise, respondents were asked if they had high cholesterol (>200 mg/dL) or normal cholesterol (<200 mg/dL). Respondents reporting high cholesterol were coded as 1 and respondents with normal cholesterol were coded as 0. BMI was measured as a dichotomous variable indicating overweight and coded as 1 if the respondent reported a BMI of 25 or over, and respondents with a BMI below 25 were coded as a 0 otherwise.

We also included independent variables representing demographic characteristics, religious sect, and experiences with stigma (heightened vigilance). Demographics characteristics were measured with five variables: age, nativity, neighborhood, education, and annual household income. Age was measured with a continuous variable. Nativity was operationalized with a binary variable, coded as 1 if the respondent was born in the United States and 0 if the respondent was born outside of the United States. Neighborhood was measured with a dichotomous variable. Respondents in urban areas were coded as 1 and those in suburban areas as 0. Annual household income was grouped into four categories: <$24,999, $25,000–$74,999, $75,000–$99,999, and >$100,000. Education was measured with a dichotomous variable: college graduate (coded as 1) and not a college graduate (coded as 0). Respondents were asked what type of Islam they identified as, and response categories included the following: Sunni, Shia, and general Islam (referent). General Islam was included for those who identified as Muslims, but did not align themselves with a sect. Internalized stigma was assessed and measured using the Abbreviated Heightened Vigilance Scale (AHVS), a scale developed for the Chicago Community Adult Study that measured how respondents prepared for or anticipated racial discrimination. Respondents were asked how often they try to prepare for possible insults from other people before leaving home, feel they always have to be careful about their appearance (to get good service or avoid being harassed), carefully watch what they say or how they say it, and try to avoid certain social situations and places. Responses ranged from 1=almost every day to 6=never, and were summed into a continuous scale ranging from 4 to 24. Lower scores indicated higher heightened vigilance.

### Analytic strategy

Univariate statistics described the characteristics of the sample. Logistic regression models estimated the relationship between health behaviors and health risks with the aforementioned controls. All models were estimated with Stata 15.0.

## Results

Sample characteristics of American Muslim women (*n*=373) are in Table 1. Two-thirds of respondents exercise according to recommendations (64.3%), 84.9% of respondents eat fruits and vegetables daily, and only 19.8% of respondents report consuming alcohol. The most common negative health characteristic in the sample was being overweight. Approximately 42% of the sample reported a BMI considered overweight (42.1%), 4.3% of respondents had high blood pressure, and 5.4% of respondents had high cholesterol.

**Table 1.** **Characteristics of American Muslim Women**

	Muslim women, *n*=373
Prevention health behaviors, *n* (%)	
Exercise	240 (64.34)
Healthy eating	314 (84.86)
Consumption of alcohol	74 (19.84)
Health risks for cardiovascular disease, *n* (%)	
High blood pressure	14 (4.26)
High cholesterol	20 (5.41)
Overweight	157 (42.06)
Demographic characteristics, *n* (%)	
Age, M (SD)	30.99 (11.14)
Marital status	
Currently married	167 (44.77)
Not married	206 (55.23)
Nativity	
U.S. born	161 (43.16)
Foreign born	212 (56.84)
Neighborhood	
Rural	17 (4.56)
Suburban	223 (59.79)
Urban	132 (35.39)
Household income	
<$24,999	50 (13.40)
$25,000–$74,999	124 (33.24)
$75,000 to $99,999	49 (13.14)
>$100,000	150 (40.21)
Education	
Not a college graduate	114 (30.65)
College graduate	258 (69.35)
Religion, *n* (%)	
Islam	
General Islam	57 (15.28)
Shia	147 (39.41)
Sunni	168 (45.04)
Heightened Vigilance Scale, M (SD)	15.09 (5.98)

M, mean; SD, standard deviation.

The age of Muslim women in the sample ranged from 18 to 69 years, with an average age of 30.1 years. Approximately 43% of the sample were born in the United States (43.2%), 44.8% were currently married, and 35.4% lived in an urban area. Over two-thirds of the respondents were college graduates (69.4%), and 40.2% reported annual household income >$100,000. About 45% of the sample identified as Sunni Muslim (45.0%), 39.4% identified as Shia Muslim, and 15.3% identified as general Islam. Respondents reported numerous instances of heightened vigilance. The average heightened vigilance scale was 15.1 (ranging from 2 to 24, lower scores reflecting more heightened vigilance). Almost two-thirds of the sample reported carefully watching what they say and how they say it a few times a month or more frequently (63.4%). Approximately 48% of the sample (47.5%) reported avoiding certain social situations and places at least a few times a month, 37.8% reported being careful about their appearance, and 21.7% of the sample tried to prepare for possible insults from other people a few times a month or more frequently (not reflected in Table 1).

Odds ratios (ORs) from the multivariate regression analysis are presented in Table 2. Age was associated with health risks, but was not associated with health behaviors. For each increase in age, the odds of having high blood pressure or high cholesterol increase by ∼1.05 (OR=1.051, *p*<0.05; OR=1.057, *p*<0.05, respectively). Likewise, for each increase in age, the odds of having high cholesterol increase by 1.08 (OR=1.080, *p*<0.001). Married respondents have 42% lower odds of exercising and 83% lower odds of drinking alcohol (OR=0.577, *p*<0.05; OR=017, *p*<0.05, respectively). Compared to foreign-born respondents, U.S.-born respondents have 2.9 higher odds of drinking alcohol (OR=2.931, *p*<0.001). Compared to respondents who have not graduated from college, college graduates high higher odds of exercising and consuming alcohol, and lower odds of high cholesterol and being overweight. College graduate respondents were associated with 2.0 higher odds of exercising and 4.9 higher odds of consuming alcohol (OR=1.956, *p*<0.001; OR=4.928, *p*<0.001, respectively). Likewise, compared to respondents who have not graduated from college, college graduate respondents have 80% lower odds of having high cholesterol and 61% lower odds of being overweight (OR=0.198, *p*<0.01; OR=0.395, *p*<0.01, respectively).

**Table 2.** **Regression Results of Behaviors and Risks Related to Noncommunicable Diseases**

	Prevention health behaviors			Risks for cardiovascular disease		
	Exercise, OR (95% CI)	Healthy eating, OR (95% CI)	Alcohol, OR (95% CI)	High blood pressure, OR (95% CI)	High cholesterol, OR (95% CI)	Overweight, OR (95% CI)
AHVS	1.023 (0.985–1.063)	1.030 (0.978–2.721)	1.085 (1.023–1.150)^***^	1.065 (0.952–1.192)	1.040 (0.952–1.136)	0.938 (0.900–0.976)^***^
Age	0.991 (0.966–1.016)	1.046 (0.998–1.097)	1.005 (0.972–1.039)	1.051 (1.004–1.101)^**^	1.057 (1.010–1.106)^**^	1.080 (1.048–1.112)^***^
Marital status						
Not currently married	Referent	Referent	Referent	Referent	Referent	Referent
Currently married	0.577 (0.336–0.991)^**^	1.178 (0.538–2.577)	0.170 (0.080–0.361)^***^	1.371 (0.339–5.544)	1.610 (0.465–5.571)	1.502 (0.847–2.580)
Nativity						
Foreign born	Referent	Referent	Referent	Referent	Referent	Referent
U.S. born	1.422 (0.873–2.318)	1.205 (0.641–2.263)	2.931 (1.484–5.787)^***^	0.473 (0.107–2.098)	2.732 (0.798–9.351)^*^	1.053 (0.642–1.728)
Dwelling						
Rural	Referent	Referent	Referent	Referent	Referent	Referent
Urban	1.087 (0.675–3.347)	0.999 (0.526–1.890)	1.341 (0.724–2.482)	0.765 (0.210–2.787)	0.772 (0.261–2.280)	1.063 (0.658–1.718)
Education level						
High school	Referent	Referent	Referent	Referent	Referent	Referent
College graduate	1.956 (1.143–3.347)^***^	1.419 (0.694–2.897)	4.928 (2.065–11.760)^***^	0.408 (0.107–1.551)	0.198 (0.061–0.646)^***^	0.395 (0.217–0.718)^***^
Household income						
<$24,999	Referent	Referent	Referent	Referent	Referent	Referent
$25,000–$74,999	1.164 (0.574–2.358)	1.827 (0.753–4.432)	1.284 (0.440–3.748)	0.173 (0.256–1.244)	1.723 (0.129–22.933)	1.108 (0.515–2.384)
$75,000–$99,999	0.743 (0.320–1.725)	2.022 (0.652–6.266)	1.793 (0.530–6.065)	1.340 (0.262–7.483)	3.432 (0.258–45.694)	1.850 (0.749–4.571)
>$100,000	1.670 (0.808–3.450)	1.208 (0.503–2.901)	1.695 (0.584–4.917)	0.168 (0.027–1.059)	4.026 (0.354–45.817)	1.108 (0.507–2.422)
Religion (Islam)						
General Islam	Referent	Referent	Referent	Referent	Referent	Referent
Shia	1.318 (0.655–2.652)	1.230 (0.456–3.320)	1.535 (0.656–3.593)^***^	0.702 (0.154–3.187)	3.446 (0.676–17.567)	1.169 (0.570–2.398)
Sunni	0.700 (0.370–1.327)	0.691 (0.300–1.594)	0.236 (0.091–0.609)^***^	0.360 (0.063–2.068)	0.858 (0.141–5.220)	1.079 (0.555–2.099)

^*^ *p*<0.1, ^**^p<0.05, ^***^p<0.01.

AHVS, Abbreviated Heightened Vigilance Scale; CI, confidence interval; OR, odds ratio.

Heightened vigilance was associated with drinking alcohol; each increase in AHVS (higher scores indicate lower heightened vigilance) was associated with a 1.09 higher odds of drinking alcohol (OR=1.085, *p*<0.01). Heightened vigilance was also associated with being overweight as each increase in the scale was associated with 0.06 lower odds of being overweight (OR=0.938, *p*<0.01); the higher the perceived stigma, the higher the odds of being overweight. Religious sect was a significant predictor of alcohol consumption. In comparison to respondents identifying as General Islam, Sunni respondents were associated with 0.76 lower odds of consuming alcohol (OR=0.236, *p*<0.01).

## Discussion

Since this study was informed by limited prior research, specific hypotheses were not proposed. Rather, this study was designed by including measures that were previously indicated as being relevant to both health behaviors (diet, exercise, and alcohol) and health risks (cholesterol, weight, and blood pressure), namely nativity (if a respondent was born in the United States or elsewhere), stigma (through heightened vigilance), and demographic characteristics. As indicated in previous studies—both with minority populations and with the general overall population—age was protective against health risks. Younger respondents had lower olds of having high cholesterol, high blood pressure, and being overweight. Advanced education was associated with better health behaviors and health risks.

More nuanced findings emerged when examining the statistical effects of stigma and nativity, traits that are relatively unique in this study population. Although the Healthy Migrant Effect suggests that being foreign born should be unilaterally protective, our results found that foreign-born individuals only had lower odds of alcohol consumption and of having high cholesterol. Although we measured alcohol consumption in this analysis, our team is aware of the challenges of interpreting such a variable with limited context and the mixed effect of alcohol (harmful and protective) depending on the degree of consumption.^37,38^ Stigma, measured through heightened vigilance, was associated with alcohol consumption and weight. Prior studies have demonstrated the harmful effects of stigma; however, our findings around stigma were mixed with increased stigma being associated with higher odds of being overweight, but lower odds of consuming alcohol. These preliminary findings contribute to the limited body of research on Muslim health. Future studies with larger sample sizes that corroborate self-report data with biomarkers are warranted.

### Limitations

Limitations should be thoughtfully considered when extending study findings. First, non-English speakers were unable to participate in this study. Although respondents were asked about their country of origin, they were not explicitly asked to declare their race or ethnicity. This is a relevant limitation, because heightened vigilance (perceived stigma) could have been compounded by stigma against race (intersectional). In addition, heightened vigilance does not capture actual experiences of discrimination. Respondents were more educated and wealthier than Americans in general, indicating a selection bias in respondents. Although online surveys have their benefits (mentioned previously), they are less likely to reach the most vulnerable, including those lacking web access. As a preliminary pilot study, these results are not necessarily generalizable, but do offer a glimmer of insight into American Muslim women's health behaviors and health profile.

## Conclusion

The rise in cardiovascular diseases is fueling a public health crisis in the United States, resulting in increased morbidity and avoidable mortality, as well as economically costing billions in healthcare.^39^ With increasing focus on precision medicine, personalized healthcare, and the promotion of patient-centered medical homes—all designed to promote prevention and assist in managing chronic health conditions—better understanding the health of understudied, often high risk, populations is imperative. Prior research indicates that examining Islam as a cultural paradigm is beneficial in that, this culture influences both health behaviors and health risks.^6,7^ Considering the overarching goals of public health and medicine are to first, do no harm, and second, to improve overall population health, more thoughtful research on populations that are hard to reach due to being stigmatized, is urgently needed to better inform healthcare policy, clinical practice, and public health prevention efforts.

## Abbreviations Used

BMI: body mass index
CI: confidence interval
HDL: high-density lipoprotein
IRB: Institutional Review Board
OR: odds ratio
SD: standard deviation

## References

[1] United States Census Bureau. Demographic turning points for the United States: Population projections for 2020 to 2060. Available at www.census.gov/library/publications/2018/demo/p25-1144.html Accessed 622, 2018

[2] The Rise of Asian Americans. Pew research center social and demographic trends. Available at www.pewsocialtrends.org/2012/06/19/the-rise-of-asian-americans/ Accessed 622, 2018

[3] U.S. Department of Homeland Security. 2007 yearbook of immigration statistics: office of immigration statistics. 2008 Available at www.dhs.gov/sites/default/files/publications/Yearbook_Immigration_Statistics_2007.pdf Accessed 622, 2018

[4] De P, Budhwani H Human papillomavirus (HPV) vaccine initiation in minority Americans. Public Health. 2017;144:86–91 28274389 10.1016/j.puhe.2016.11.005

[5] Budhwani H, De P Disparities in influenza vaccination across the United States: variability by minority group, Asian sub-populations, socioeconomic status, and health insurance coverage. Public Health. 2016;138:146–153 27178130 10.1016/j.puhe.2016.04.003

[6] Budhwani H, Anderson J, Hearld KR Muslim women's use of contraception in the United States. Reprod Health. 2018;15:1–8 29304829 10.1186/s12978-017-0439-6 PMC5756427

[7] Budhwani H, Hearld KR Muslim women's experiences with stigma, abuse, and depression: results of a sample study conducted in the United States. J Women's Health (Larchmt). 2017;26:435–441 28263695 10.1089/jwh.2016.5886

[8] Herner A UChicago researcher documents dramatic shortcomings tracking American Muslim health disparities. Available at https://sciencelife.uchospitals.edu/2015/04/06/uchicago-researcher-shows-dramatic-shortcomings-tracking-american-muslim-health-disparities Accessed 622, 2018

[9] Haque A Religion and mental health: the case of American Muslims. J Relig Health. 2004;43:5–58

[10] Walpole SC, McMillan D, House A, et al. Interventions for treating depression in Muslim patients: a systematic review. J Affect Disord. 2013;145:11–20 22854098 10.1016/j.jad.2012.06.035

[11] My Life Check—Life's Simple 7. American Heart Association website. 2017 Available at www.heart.org/HEARTORG/Conditions/My-Life-Check-Lifes-Simple-7_UCM_471453_Article.jsp#.WAfVEKOZMy4 Accessed 1214, 2017

[12] Morales LS, Kingston RS, Valdez RO, et al. Socioeconomic, cultural, and behavioral factors affecting Hispanic health outcomes. J Health Care Poor Underserved. 2002;13:477–503 12407964 10.1177/104920802237532 PMC1781361

[13] Kennedy S, McDonald JT, Biddle N Australian Bureau of Statistics and Centre for Aboriginal Economic Policy Research. The healthy immigrant effect and immigrant selection: Evidence from four countries. 2006 Available at https://pdfs.semanticscholar.org/75ab/d0284c629f564c2294c54929ea4d6995d441.pdf Accessed 713, 2017

[14] Budhwani H, Hearld KR, Chavez-Yenter D Generalized anxiety disorder in racial and ethnic minorities: a case of nativity and contextual factors. J Affect Disord. 2015;175:275–280 25661302 10.1016/j.jad.2015.01.035

[15] Budhwani H, Hearld KR, Chavez-Yenter D Depression disparities in racial and ethnic minorities: the impact of nativity and discrimination. J Racial and Ethn Health Disparities. 2015;2:34–42 26863239 10.1007/s40615-014-0045-z

[16] Hearld KR, Budhwani H, Chavez-Yenter D Panic attacks in minority Americans: the effects of alcohol abuse, tobacco smoking, and discrimination. J Affect Disord. 2015;174:106–112 25496758 10.1016/j.jad.2014.11.041

[17] Singh G, Miller B Health, life expectancy, and mortality patterns among immigrant populations in the United States. Can J Public Health. 2004;95:I14–I21 15191127 10.1007/BF03403660 PMC6975697

[18] Cunningham A, Ruben J, Narayan K Health of foreign-born people in the United States: a review. Health Place. 2008;14:623–635 18242116 10.1016/j.healthplace.2007.12.002

[19] Akresh IR Dietary assimilation and health among Hispanic immigrants to the United States. J Health Soc Behav. 2008;48:404–417 10.1177/002214650704800405 18198687

[20] Goel MS Obesity among US immigrant subgroups by duration of residence. JAMA. 2004;292:2860–2867 15598917 10.1001/jama.292.23.2860

[21] Hajj E, George D Health Behaviors and Health Outcomes of Arab Americans: A Socio-ecological Perspective. Ann Arbor, MI: University of Colorado Health Science, 2012

[22] Brittin HC, Obeidat BA Food practices, changes, preferences and acculturation of Arab students in US universities. Intl J Consum Stud. 2011;35:552–559

[23] Hussaini MM Why halal haram lists do not work? Sound Vision website. Available at www.soundvision.com/article/why-halal-haram-lists-do-not-work Accessed 622, 2018

[24] Stodolska M, Livengood J The influence of religion on the leisure behavior of immigrant Muslims in the United States. J Leis Res. 2006;38:293–320

[25] Caperchione CM, Kolt GS, Mummery WK Physical activity in culturally and linguistically diverse migrant groups to western society. Sports Med. 2009;39:167–177 19290674 10.2165/00007256-200939030-00001

[26] Pollard TM, Guell C Assessing physical activity in Muslim women of south Asian origin. J Phys Act Health. 2012;9:970–976 22971888 10.1123/jpah.9.7.970

[27] O'Driscoll T, Banting LK, Borkoles E, et al. A systematic literature review of sport and physical activity participation in culturally and linguistically diverse (CALD) migrant populations. J Immigr Minor Health. 2013;16:515–530 10.1007/s10903-013-9857-x 23771744

[28] Wray S Connecting ethnicity, gender, and physicality: Muslim Pakistani women, physical activity and health. In: Gender and Sport: A Reader. Edited by Flintoff A, Scraton S New York: Routledge, 2002, pp. 127–140

[29] Katulanda P, Jayawardana R, Ranasinghe P, et al. Physical activity patterns and correlates among adults from a developing country: the Sri Lanka diabetes and cardiovascular study. Public Health Nutr. 2012;16:1684–1692 22995708 10.1017/S1368980012003990 PMC10271739

[30] Begam NS, Srinivasan K, Mini GK Is migration affecting prevalence, awareness, treatment and control of hypertension of men in Kerala, India? J Immigr Minor Health. 2016;18:1365–1370 26860477 10.1007/s10903-016-0353-y

[31] Veenstra G, Patterson AC South Asian-White health inequalities in Canada: intersections with gender and immigrant status. Ethn Health. 2016;21:639–648 27133779 10.1080/13557858.2016.1179725

[32] Tailakh A, Mentes JC, Morisky DE, et al. Prevalence, awareness, treatment, and control of hypertension among Arab Americans. J Cardiovasc Nurs. 2013;28:330–337 22722474 10.1097/JCN.0b013e31825638ae PMC4445860

[33] Ujčič-Voortman JK Ethnic Disparities in Cardiovascular Disease Risk: The Distribution of Risk Factors among Amsterdam Residents with a Turkish and Moroccan Ethnic Background. Amsterdam: Vrije Universiteit, 2011

[34] Glenday K, Kumar B, Tverdal A, et al. Cardiovascular disease risk factors among five major ethnic groups in Oslo, Norway: the Oslo immigrant health study. Eur J Cardiovasc Prev Rehabil. 2006;13:348–355 16926663 10.1097/01.hjr.0000214616.14361.51

[35] Daryani A, Berglund L, Andersson A, et al. Risk factors for coronary heart disease among immigrant women from Iran and Turkey, compared to women of Swedish ethnicity. Ethn Dis. 2005;15:213–220 15825967

[36] Wright KB Researching Internet-based populations: advantages and disadvantages of online survey research, online questionnaire authoring software packages, and web survey services. J Comput Mediat Commun. 2005;10 DOI:10.1111/j.1083-6101.2005.tb00259.x

[37] Sternthal MJ, Slopen N, Williams DR Racial disparities in health. Du Bois Rev. 2011;8:95–113 29887911 10.1017/S1742058X11000087 PMC5993442

[38] Williams DR Discrimination Resource. Harvard University website. Available at http://scholar.harvard.edu/davidrwilliams/book/export/html/32495 Accessed 622, 2018

[39] Alcohol and Heart Health. American Heart Association website. Available at www.heart.org/HEARTORG/HealthyLiving/HealthyEating/Nutrition/Alcohol-and-Heart-Health_UCM_305173_Article.jsp#.WyVEAdVKiUk Accessed 621, 2018

